# Introduction to emerging industrial applications of cannabis (*Cannabis sativa* L*.*)

**DOI:** 10.1007/s12210-021-00979-1

**Published:** 2021-03-19

**Authors:** Giuseppe Sorrentino

**Affiliations:** 1 CNR-Institute for Sustainable Plant Protection, Piazzale Enrico Fermi, 1, 80055 Portici, NA Italy; 2 CNR- Institute for Mediterranean Agriculture and Forest, Ercolano, NA Italy

**Keywords:** *Cannabis sativa* L., Green economy, New supply chain, Nutraceutical food, Green building, Textile industry, Pharmaceutical

## Abstract

The Italian Law of 22 November 2016 has legalized the cultivation of hemp, which drives the development of sustainable agriculture by generating new products with high added value in the new context of circular economy. Hemp cultivation is known for its low environmental impact, as hemp grows fast, suppresses weeds and does not need pesticides. It has no specialized parasites, favors pollination and improves the physical and chemical soil fertility. Recently, many countries have increased their interest in hemp (*Cannabis Sativa *L.), considering it as a climate-friendly crop that can mitigate climate change and desertification. For these reasons, hemp can be a new protagonist of Italian agriculture already oriented towards the objectives of EU 2030 which predicts 40% decrease in greenhouse gas emissions compared to 1990. The hemp cultivation can activate a new supply chain by allowing using different parts of the plant, benefiting farmers, environment, and human health. Indeed, although a very old plant, hemp will be one of the main protagonists of the green economy in the near future. Its seeds can be used by agri-food industry to produce flour, pasta, pastry and oil, while the stem through *canapulo* (woody part of stem) in green building sector. Its fiber (external part of stem) will find new applications in textile industry. As for its inflorescences and roots, thanks to the extraction of bioactive molecules, they will play an important role in the pharmaceutical and parapharmaceutical industry. Finally, only the medical sector with Δ^9^‐tetrahydrocannabinol (THC) extraction from inflorescence is not yet regulated by the aforementioned Italian Law.

## Historical and legislative background of hemp cultivation in Italy

Hemp (*Cannabis Sativa* L.), native to the Himalayas, has spread to India and the Far East since the Neolithic, and China is the country where it is cultivated for the longest time. Its introduction in Europe probably dates back to the second millennium BC. In Italy, it is reported in the first century BC, and was found in many Etruscan findings. Later, it was used by the Roman Empire for the construction of sails, nets and ropes, but saw a broader diffusion only in the Middle Ages especially in the Po Valley. In the following centuries, the cultivation extended in great part of Central Italy up to Campania and Basilicata. In the twentieth century, after having recorded a progressive increase in the cultivated area up to 90,000 ha, from the 1940s throughout the 1960s, it experienced a decline and almost disappeared due to the introduction of synthetic fibers and especially due to Italy’s adhesion to the Protocol on the Single Convention on Narcotic Drugs proposed by the United Nations, signed in New York on 30th March 1961 and subsequently to the Protocol of Amendment adopted in Geneva on 25 March 1972. This amendment was transformed by Italian Government in law through the publication in the Official Journal number 236 of 10 September 1974.

Only since the beginning of the twenty-first century, the interest for hemp has revived thanks to the Community Legislation and especially the Council Regulation 1251/1999/EC of 17/05/1999 which established a support scheme for producers of certain arable crops, including industrial hemp, and was followed by subsequent implementing regulations, the 2316/1999 and 327/2002, of the European Commission and the Regulation 953/2006 of the European Council on the introduction of aid for the processing of flax and hemp grown for fiber. But only with the Regulation (EU) No. 1307/2013 of the European Parliament and the Council of 17 December 2013 (last reform of the CAP 2014–2020), hemp cultivation was included among those eligible for CAP payments on the condition that the seed used for the cultivation are from registered varieties in the European catalog with a content of Δ^9^‐tetrahydrocannabinol (THC) less than 0.2%. In Italy, the application of the Community Regulations on the cultivation of industrial hemp has precise legislative reference (Law 242, 2016), valid from January 2017, which has among its purposes: "Support and promotion of the hemp sector (*Cannabis sativa* L.) as a crop capable of reducing environmental impact in agriculture, reducing land consumption and desertification and loss of biodiversity, and as a crop to be used as a possible substitute for surplus crops and as a rotational crop." The same law also provides an exemption of liability for the farmers in case that the THC content is more than 0.2%, but less than 0.6%, and also encourages the development of certain sectors such as green building, food and textiles. But it does not regulate the possible use of inflorescences for the pharmaceutical and parapharmaceutical sectors.

### Importance of hemp cultivation for environment and agriculture

All of us who work in the field and ordinary citizens can begin to argue about the topical of this ancient culture starting with a simple question: *Why today we need to encourage the cultivation of hemp in the world?* The answer is because hemp acts positively both on ecosystem and human health, thus promoting the many ecological and biological mechanisms that govern the conservation and evolution of life itself.

In fact, its ecological profile is perfectly oriented in the containment directions of the so-called Planetary Boundaries that are the limits of sustainable ecological development proposed by the 'Earth System and Environmental Scientist led by Johan Rockström from the Stockholm Resilience Center and Will Steffen from Australian National University for Planet Earth (2009). The Stockholm Resilience Center for Planet Earth is an international study center that deals with the sustainable development of the planet earth based on the use of natural resources. The "planetary limits" traced in 2009 by the Swedish researcher define the boundaries within which we human beings can operate safely, without harming the balance of the planet.

Three of these limits have already been largely exceeded and are at a point of no return. The ecological processes that can positively interfere with some of these limits are those in which the cultivation of hemp can generate great benefits to environment. The first concern is that the CO_2_ concentration is currently standing at 450 parts per million (ppm) in the atmosphere, much more compared to the pre-industrial value of 280 ppm. Hemp acts positively on the concentration of CO_2_ in the atmosphere because it is a culture ΔCO_2_ negative. It means that this crop is capable in the carbon cycle of removing more CO_2_ from the ambient than it emits. The high ability to sequester CO_2_ from the atmosphere is a trait common to many agricultural species, especially woody ones, but this feature is particularly evident in hemp which has a very rapid growth compared to other herbaceous crops. For example, hemp compared to other fiber crops such as cotton and flax which have a longer growth cycle produces about 8 and 3 times more biomass, respectively. It should be noted that the CO_2_ values absorbed by 1 hectare of hemp vary considerably according to the agronomic practices adopted and the biomass produced per hectare. Melosini (2017) for a production of biomass produced between 8 and 12 tons report values of CO_2_ seized between 10 and 15 tons per hectare. Adesina et al. (2020) instead have related the increase in dry matter of the stem (where 80% of atmospheric carbon is sequestered and stored) as a function of nitrogen fertilization for nitrogen values between 0 and 120 kg/ha with the possibility of sequester up to 22 tons of CO_2_ per hectare. This great variability of hemp to sequester carbon depends on the fact that the increase in the stem per unit of nitrogen cannot be accurately determined from the literature data due to the different methods used and the cultivation environment. In addition, recent agronomic research has shown that hemp can also have a beneficial effect on the reduction of agricultural greenhouse gases through the mechanisms that govern its nitrogenous nutrition. In fact, it has recently been shown that hemp cultivation has a better vegetative development and better seed quality if slow release fertilizers such as UREA are used compared to synthetic fertilizers such as ammonium nitrate (NH_4_NO_3_) (Tedeschi et al. 2020).

The ammonium nitrate also compared with UREA, has favored, during most of the vegetative cycle of hemp, a greater emission of greenhouse gases in the atmosphere such as nitrous oxide (N_2_O) which is extremely dangerous because it heats 297 times more than CO_2_ and alone it accounts for 7% of total Italian emissions (IPCC Report 2018)*.*

The low efficiency in the use of nitric nitrogen is an eco-physiological characteristic of this crop when compared with other interesting species from the point of view of the bioeconomy such as cotton and kenaf. In fact, it has been shown that photosynthetic metabolism of hemp in relation to the foliar nitrogen content is more efficient at low nitrogen levels (< 2 g N/m^2^) than cotton and kenaf; while above this threshold value, it is lower compared to the other two species (Tang et al. 2017). The consequence is that the slow absorption of nitric nitrogen can favor both the leaching processes with pollution of the groundwater and denitrification with a consequent increase in the emission of the greenhouse gas N_2_O into the atmosphere. For these two combined effects, namely the seizure of atmospheric CO_2_ in its own biomass and lower N_2_O emissions into the atmosphere due to the favorable use of slow release nitrogen fertilizers, hemp can reasonably be considered a *climate-friendly* crop, namely to combat climate change.

Finally, hemp also has positive effects on the containment of biodiversity loss, another parameter of the Planetary Boundaries (number of extinct species /million species/year) whose limit has also exceeded the point of no return and which is currently > 100. The proposed value to counter biodiversity loss should be around 10. The increase in plant biodiversity linked to the cultivation of hemp depends on the fact that, thanks to the strong emission of terpenic essences from its inflorescences, hemp attracts pollinating insects. The crop is also itself a source of biodiversity because its pollen carried by the wind can reach a considerable distance of 3 km (Campiglia 2020).

**Fig. 1:**
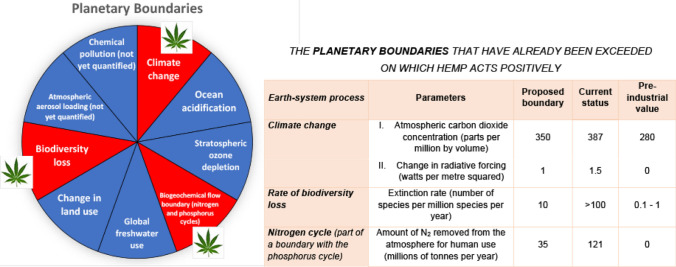
Beneficial effects of hemp on the three limits of the Planetary Boundaries proposed by Johan Rockström from the Stockholm Resilience Center and Will Steffen from Australian National University for Earth Planet (2009) that have passed the point of no return. *Cannabis Sativa* L. acts positively on C0_2_ Air Concentration, Nitrogen Cycle and Biodiversity loss. The leaf symbol in red area is referred to the Planetary Boundaries that is positively influenced by hemp and reported in the table. Figure  was revised after Rockström et al. (2009)

Moreover, thanks to its high size and the fact that it is not weeded, it creates a microclimate near the inflorescences that is favorable to pollinating insects. A very recent research conducted in the USA has highlighted that hemp, thanks to its flowering cycle that occurs at a different time compared to other species and also to the abundant pollen produced, has favored the nutrition of various species of bees, increasing their biodiversity. Thanks to the increase in pollination with other crops in the area, the biodiversity of the entire agro-ecosystem under study has also been increased (Flicker et al. 2020). The beneficial effects of hemp on the environment are shown in Fig. 1.

Moreover, territorial researches in the last 20 years have identified some indices of prediction of the desertification processes in existence and it has been demonstrated that 41% of the soils of Southern Italy are currently subject to desertification phenomena (Perini et al. 2008). In many of these areas, new cultivation systems are being studied with the introduction of industrial *non-food* crops to try to maintain productive agricultural soils otherwise subject to abandonment and desertification.

**Fig. 2:**
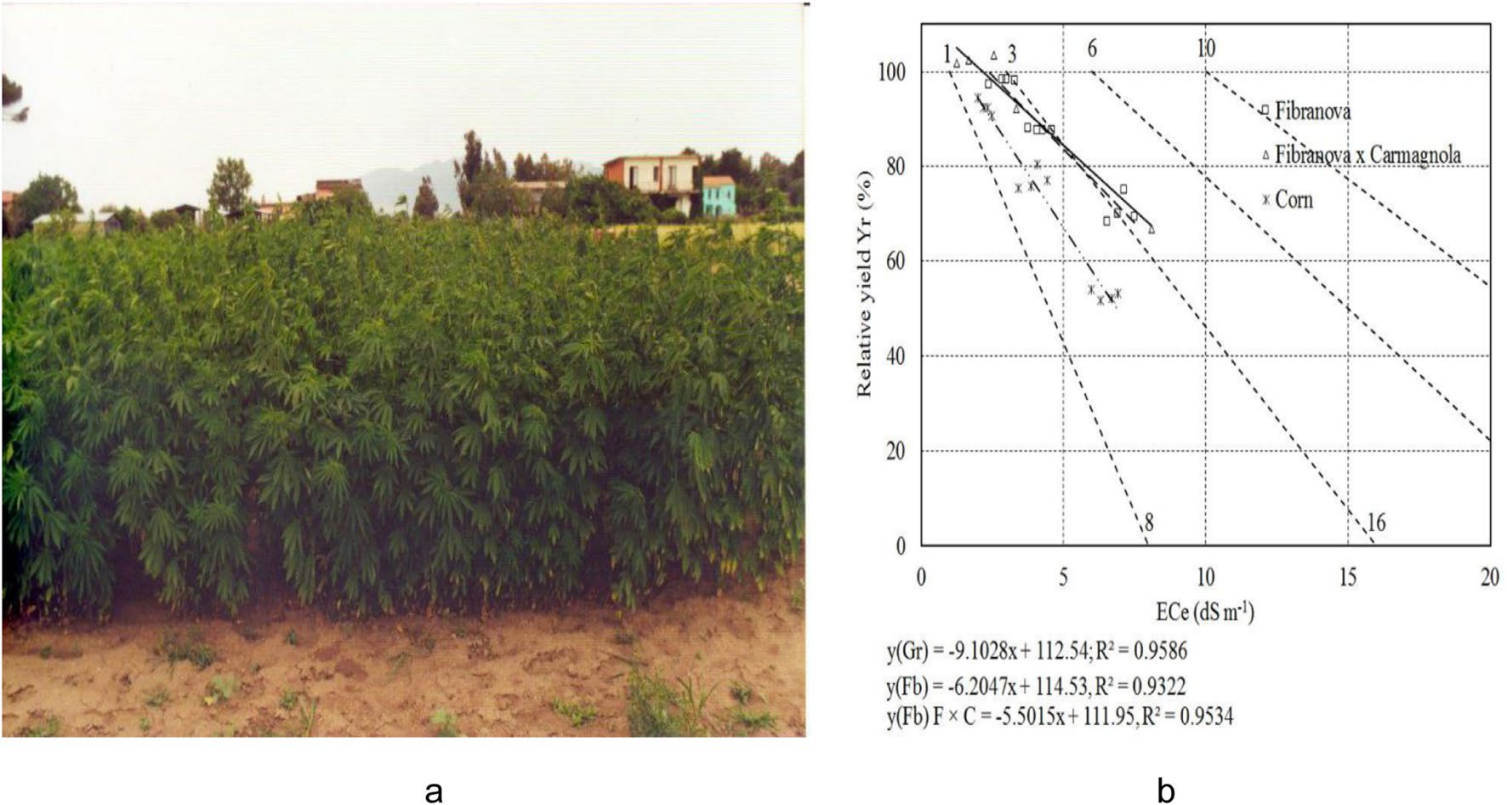
Experimental plot of *Cannabis Sativa* L. for fiber (a) and comparison of relative yield between silage grain maize and fiber hemp (cv. Fibranova and cv. Fibranova × Carmagnola) in relation to soil conductivity (ECe) (b). Agronomic trial tests were carried out in the Volturno Plain at the experimental farm in Vitulazio (CE), of the CNR- Institute of Agricultural and Forestry Systems in the Mediterranean located in Ercolano (NA) in 2000 (a) and 2002 (b). Unpubblished data of 2002 courtesy of Dr. Anna Tedeschi CNR-IBBR

The hemp fiber together with other industrial crops has been tested in the last 15 years in salinized soils of the Volturno Plain in the North of the Campania Region (Sorrentino et al. 2014). This area was once a fertile alluvial plain and gave rise to almost all the fruit and vegetable production of imperial Rome whereas today is subject to high soil desertification due to the combined effect of climate change and human activities. The reduction of rainfall caused by climate change has led to greater exploitation of the groundwater by local farmers who begin to find, given the proximity to the coast, increasingly saline water and unsuitable for irrigation (Pagliuca 2004). In this context, from a productive comparison between fiber hemp and maize on saline soil with a high electrical conductivity (Ece), it was found that, at a value of 8 ds/m Ece, maize showed a reduction in grain production by 50%, while fiber hemp at the same value of Ece showed a reduction of the fiber produced less, equal to 25% (Tedeschi et al. 2003). These production reductions were calculated and normalized in accordance with the Maas and Hoffmann report (1977) which measured the loss of agricultural production in tons per hectare (Ton/Ha) in relation to the degree of salinization of the soil (Ece) compared to the maximum achievable in non-salinized soils. Even at more moderate values of soil electrical conductivity equal to 4.5 ds/m, fiber hemp was more productive than other renewal crops such as sunflower, containing the reduction within 10% compared to 15% of sunflower (Sorrentino et al. 2000). Figure 2 shows an experimental plot of fiber hemp (a) and comparison on a highly salinized soil between maize and two varieties of fiber hemp (Carmagnola and Fibranova × Carmagnola).

We can, therefore, conclude that hemp is very important for the protection of the environment and agriculture because it can combat climate change and desertification processes in a positive and balanced manner. For these reasons, hemp can play a very important role in agriculture in the near future and be, especially in the current process of green transition, an active player in the green economy considering that the agro-industrial sectors that it can activate are already naturally "green oriented" (Sorrentino et al. 2019).

## Hemp and food chain

The agri-food chain derived from hemp can be the biggest innovation for an Italian-made food product industry, which is a 50-billion-euro export industry and is considered as one of the pillars of the Italian economy. Since 2017, the demand for hemp based food has grown by 500%. Foods that can be made from hemp seed include bread, pizza, oil, beer, milk, chocolate, ice cream and snacks. Hemp seeds, which often enrich healthy salads, can also be used to make a versatile flour useful in the production of different foods. Hemp flour has 21% less calories than a 00-flour made from traditional cereals, and a bitter and rustic taste, like whole meal. It is also gluten free, so it is a great alternative for people living with celiac disease. Hemp seeds have 25% protein of which 65% is edestin. Recent research has studied the amino acid profile of this globulin (Docimo et al. 2014), and the data obtained show that edestin is able to provide, similar to animal proteins, eight essential amino acids, that are derived from one’s diet. In addition, hemp as a protein crop can play a very important role in world food security as a new source of plant proteins which can reduce the consumption of animal proteins. Hemp oil is obtained with cold pressing from hemp seeds. Monserrat-de la Paz et al. (2014) highlighted its composition. It contains about 87% unsaturated fatty acids, of which 55–57% linoleic acid (LA), *ω*−6 series; 16% alpha-linolenic acid (ALA), *ω*−3 series; 2,7% gamma-linolenic acid (GLA), *ω*−6 series; 0.8% stearidonic acid, series *ω*−3; and 11% oleic acid. The most important quality of this oil is undoubtedly the presence of essential polyunsaturated fatty acids Omega 6 and Omega 3 in the best proportion for the human being: no other food in nature is in fact able to guarantee a proportion 3:1, ratio recommended by medical research and the most advanced theories in the field of nutrition. In addition, the quality of the fats present in hemp oil measured through the indices of atherogenic IA (< 0.31) and thrombogenicity IT (< 0.20–0.57) (Siano 2017) are excellent for human health and indicate a low tendency to form atherosclerosis in relation to a consumption of foods rich in saturated fatty acids. Hemp oil is rich in polyphenolic compounds (Andre et al 2016), and clinical and nutritional studies show that it prevents many diseases including cancer, neurodegenerative diseases, and gastrointestinal disorders (Zang and Tsao 2016). Another benefit to is that it hemp is vegan friendly and plant-based protein. Most current plant-based proteins on the market are made of pea protein or rice protein, hemp can be used in replacement of these. In regards to hemp oil, it is a source of fatty acids (EFAs) and relatively “allergen” friendly including gluten free. Beyond celiac disease mentioned, there are other conditions such as Hashimoto’s thyroiditis that benefit from gluten-free diets, and hemp could be used in protein based powders. It is a beneficial alternative for people with other food intolerances to dairy and soy. Hemp is a plant source of Vitamin D, and in regards to COVID -19, we have seen an increased need for Vitamin D as it has shown to be protective against worse outcomes (Grant et al. 2020). Hemp flour is a waste product derived from hemp oil production. It is rich in fiber, microelements and phytosterols that limit the accumulation of normal cholesterol. High quantities of beta-sitosterol of 528 mg/kg were reported by Siano et al. (2019) in the cultivar of a specific hemp variety Fedora. A daily dose of 2 g of phytosterols can reduce cholesterol uptake in the diet by 40–50% with a decrease in serum cholesterol by 6–10%. Finally, another aspect to be considered within this chain concerns the presence in hemp flour of anti-nutrient substances such as inhibitors of trypsin or phytic acid that can inhibit the absorption of iron in human nutrition. Recent studies are trying to detect cultivars with low amounts of antinutritional factors (Galasso et al. 2017) or by setting up research projects through biotechnology to intervene on the most promising ones from the nutritional point of view, eliminating or minimizing the presence of these factors. Actually, the development of hemp agri-food chain seems the most promising because it does not require large economic investments compared to others but currently has two limits that have not allowed the full exploitation of hemp. The first is represented by a lack of standardized production process that can attest to the high quality and shelf-life of the final products. This is especially true for hemp oil, very unstable and easily subject to rancidity processes due to the high level of unsaturated fats. The second limit instead comes from the possibility of finding traces or small amounts of tetrahydrocannabinol (THC) in foods, that the circular of the Ministry of Health of 22nd May 2009, on the instruction of the Higher Institute of Health, has admitted "it is possible to detect in flour and oil traces of THC due exclusively to contamination of floral organs and the adoption of unsuitable practices of seed screening". Unfortunately, this technical aspect has not yet been fully clarified by the recent Decree (Official Journal OJ 15/1/2020) which defined the maximum amount of THC allowed only for foods listed in Table 1 and not for other foods and beverages such as beer, herbal teas, cakes and biscuits.

**Table 1:** Total admissible maximum tetrahydrocannabinol (THC) concentrations according to the Decree of 4th November 2019 of the Ministry of Health in foods derived from hemp published in the Official Journal of 15th January 2020

Total admissible maximum tetrahydrocannabinol (THC) concentrations according to the decree of Ministry of Health Italy	
Foods	Maximum Limits
Hemp seeds Flour obtained from hemp seeds	2.0 mg/Kg
Oil obtained from hemp seeds	5.0 mg/Kg
Supplements containing foods derived from hemp	2.0 mg/Kg

From an investigation conducted on hemp beer by Piscitelli et al. (2017), no amounts of THC were detected (although hemp inflorescences were also used in the production process), but only small traces of other non-psychotropic cannabinoids. On the basis of these results and current legislation, this beer could be considered safe for consumers, but the law has not defined the minimum THC limits in this drink for it to be put on the market. This legislative uncertainty, given that process and product innovations can be targets easily reached by the Italian food industry, remains for now the only limit to the full exploitation of the agro-food chain of hemp in Italy.

## Hemp and green building sector

The development of green building is one of the fundamental issues and can no longer be postponed in the path of green transition, because construction along with transportation and agriculture are three sectors that affect climate change the most. Since 1992, from the Rio de Janeiro Conference, emissions have risen from 21 billion CO_2_ equivalent to the current 40 billion: the tumultuous development of the construction sector, especially in countries like China, has had a huge impact on the increase in emissions. In Italy, the construction sector has an economic value of 237 billion of euros and alone produces 30% of total CO_2_ emissions. The goal of zero use of fossil fuels by 2050, therefore, suggests also a profound change in the current construction technologies to build nearly zero energy buildings (*NZEB*s), namely buildings with almost zero energy consumption. To achieve this, it is necessary to focus on the new materials and technologies needed to produce them that require low amounts of energy (LCA-*Life Cycle Assessment*) throughout their life cycle. The environmental impact of hemp can be estimated through a Life Cycle Analysis (LCA), a method that allows the study of the potential impact of a product by quantifying and evaluating the resources used and the environmental emissions at each stage of the life cycle: analysis starts from the point of extraction of these resources, to the production of materials and the final product, to the use of the product over time and to its final disposal and recycling (Guinée 2002). Hemp used in green building is a renewable material and throughout the production cycle, from biomass produced in the field up to the construction of the block lime/hemp and plaster, is involved in technological processes characterized by low carbon emissions. In green building, the stem of hemp, called *canapulo,* is used and can store CO_2_ both as a starting material and as a finished product. The stem can be suitably shredded, pressed and used as a panel for partitions in civil dwellings. The *canapulo* can also be processed and mixed with other materials and wood-based structures that improve its performance. In fact, the materials obtained for the green building from hemp are very light, inert to moisture and heat and therefore favor the maintenance of a constant temperature by reducing the use of heating and air conditioners (Aversa et al. 2019). The shredded *canapulo* can also be mixed with lime to produce plasters.

In a recent study, it has been shown that from the production of *canapulo* up to the production of the lime–*canapulo* block and to the realization of one square meter of a wall of a building suitably plastered with a mixture of lime–*canapulo*, the wall built with these materials is able to lower the *carbon footprint* of about 60 kg CO_2_ equivalent compared to other materials (Arrigoni ET AL. 2017). From the agronomic point of view, the most suitable hemp varieties for green building should have a relatively long life cycle and with a *canapulo* ratio of more than 70% of the plant material compared to fiber. The density of plants must be limited with spaced crops to promote the growth of the stem because by increasing the density of plants, the stem does not branch and is produced a greater amount of primary fiber than the *canapulo*. Since the green building sector is a new sector, there are currently no varieties suitable for green building, so it is necessary to refer to those allowed by the Plant Variety Database (Delegated Regulation 639–2014) which integrates Regulation (EU) 1307/2013. Monoecious cultivars, however, seem more suitable for this purpose because they have more robust plants than dioecious ones (Campiglia 2020). Investigations on the structure of the seeds and *canapulo* in 3D to identify those most suitable for seed production or green building were initiated by Gargiulo et al. (2017).

**Fig. 3:**
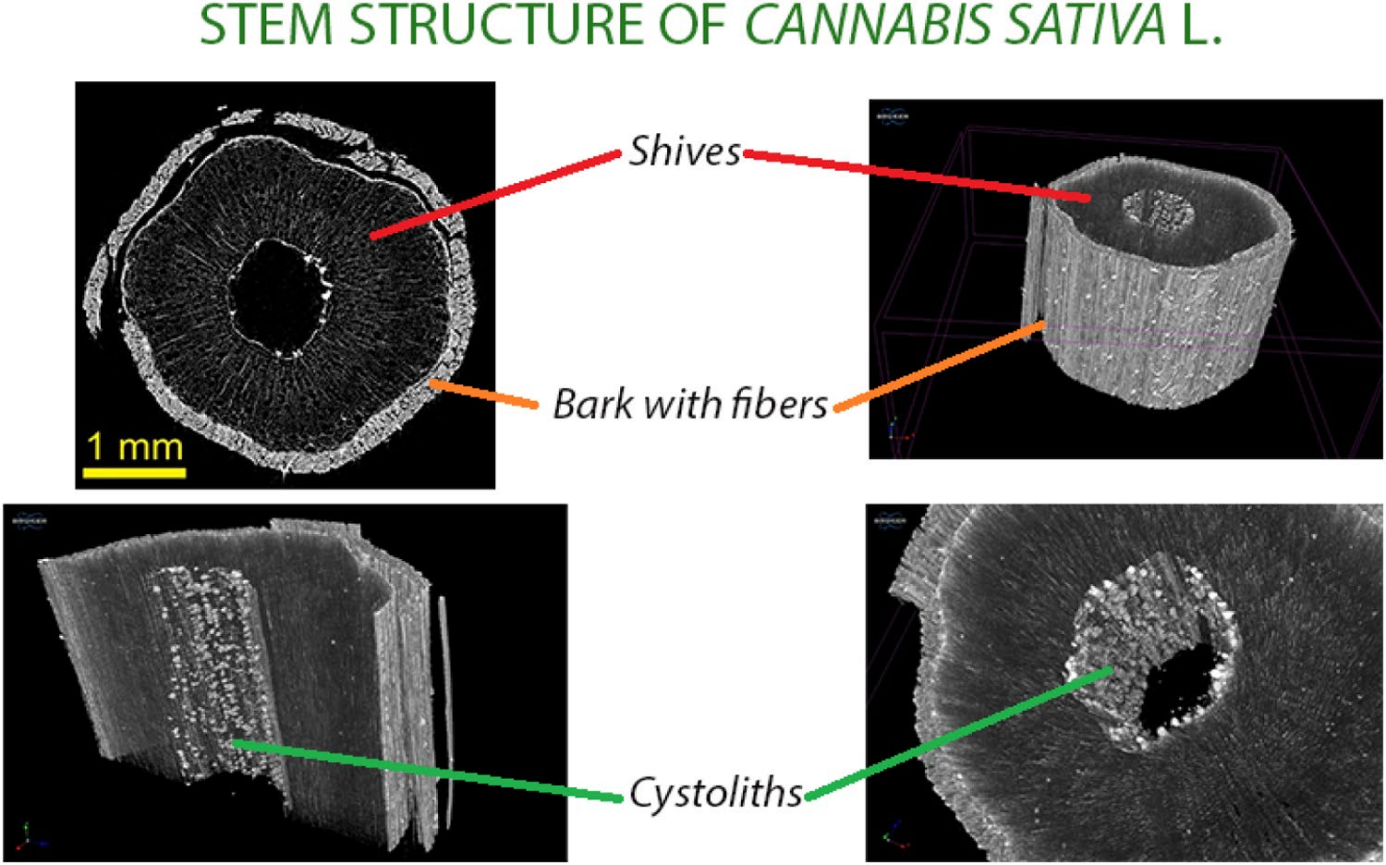
The 3D structural survey of the characteristics of stem of *Cannabis sativa* L. cultivar Uso 31 for an evaluation of the hemp/fiber ratio. By kind permission of Dr. Giacomo Mele responsible for the CNR-Isafom X-ray Microtomography Laboratory

**Fig. 4:**
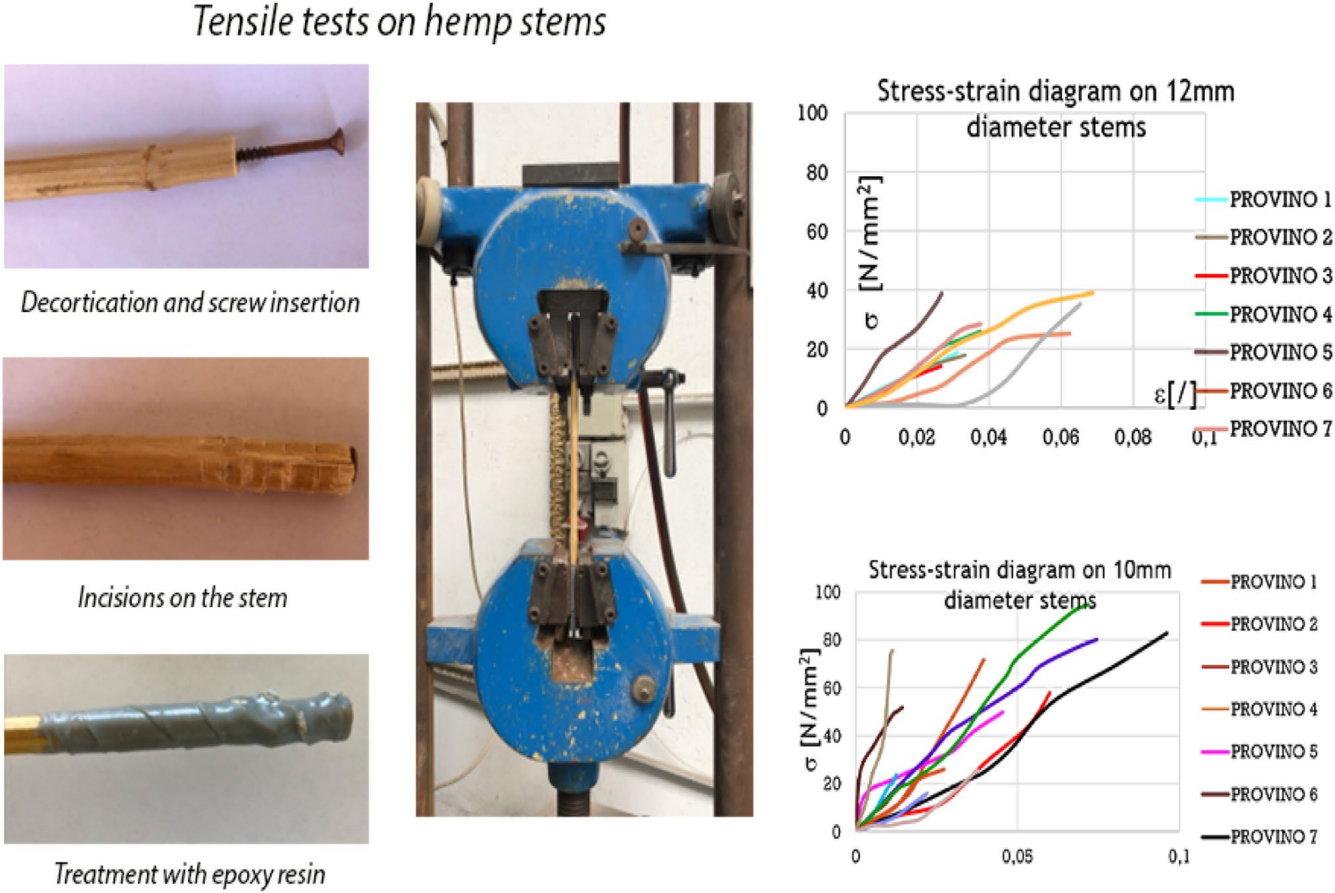
Test of rupture of the hemp stem (courtesy of Prof. Antonio Formisano of the Polytechnic School and Basic Sciences—(Department of Structures for Engineering and Architecture, University Federico II of Naples)

There are varieties such as the Futura 75 that can be dual-purpose, i.e., used both to produce *canapulo* and seed. Figure 3 shows some visualizations of the structure of the hemp stem in 3D in which are visible the *canapulo* and the bark with fibers both external and internal and is possible to identify the ratio *canapulo*/fiber. The long fiber is obtained from the outer part of the *canapulo* (between 5 and 55 mm) that can have different uses in the textile sector but is also used in green building thanks to its great tensile strength. It can be used to arm arches and wooden beams by increasing the breaking resistance. Very recently, tensile tests on hemp stems were conducted at the Department of Structures for Engineering and Architecture of the University of Naples Federico II. Once the problems of amortization have been solved to avoid the fraying of the hemp stem margins in the vice, the results on 10 mm diameter stems showed the best results, with average strength and ultimate strain values about 1/3 and 1/2 of steel ones, respectively. Such results, through a more industrialized process, could improve much more, allowing to the hemp stems to replace steel meshes as reinforcing system of existing structures (Formisano and Davino 2020) (Fig. 4).

From the economic and social points of view, in Italy, the green building chain could have a great development considering that the damages in Central Italy caused by the earthquakes and the restoration of some public works, whose materials have finished the life cycle and need a rapid and urgent restructuring. The limits for the development of this supply chain are mainly linked to the reduced agricultural area invested in hemp. This means the lack of availability of raw materials to make continuous work of the plants whose realization requires a great economic investment. In addition, the transport of the first processing material of hemp is very expensive for most italian farmers because the two first processing sites are located in Carmagnola (TO) and Crispiano (TA) therefore very far from all the companies operating in much of Italy. It would, therefore, be necessary to have at least two or three new processing sites in Central Italy to facilitate the takeoff of this important sector. Moreover, for a full activation of this new sector, there must necessarily be a strong synergy between the public and private sectors. An example of this synergy is on the basis of the pilot project "The bio district of Hemp in Irpinia" which is currently being evaluated by the European Commission with the involvement of all research entities operating in Campania, and 25 municipalities in the Irpinia area that have signed an agreement for the development of green building linked to the cultivation of hemp in the area (http://www.borghinrete.it/2017/04/19/convegno-su-il-biodistretto-della-filiera-della-canapa-in-irpinia/). In this project, in which several companies in the sector are also involved, it is planned to build a new processing site for the treatment of hemp straw to be carried out in a small city, Vallata (AV), Southern Italy.

## Hemp and textile sector

The Italian fashion exports are worth 28 billion euros and are the second only following the food sector (50 billion euros). The recovery of the textile industry thanks to hemp would have a big impact on the use of innovative and green materials derived from this sector that has always had a great tradition in Italy. The textile industry, in fact, during the autarchic period, had at its disposal an area of about 90,000 hectares and the yarns produced in Italy were considered the best in the world. All this because the research was at the forefront thanks to the Textile Corporation and the establishment of the National Hemp Consortium (*Consorsio Nazionale Canapa*—CNC) which experimented new production techniques, such as green scutching with non-macerated plants, that allowed an increase in fiber yield per hectare, and new techniques for processing hemp fibers, that have increased their yarn obtaining a raw material intended for easier processing with other fibers and on existing installation (Ruzzenenti 2017).

**Fig. 5:**
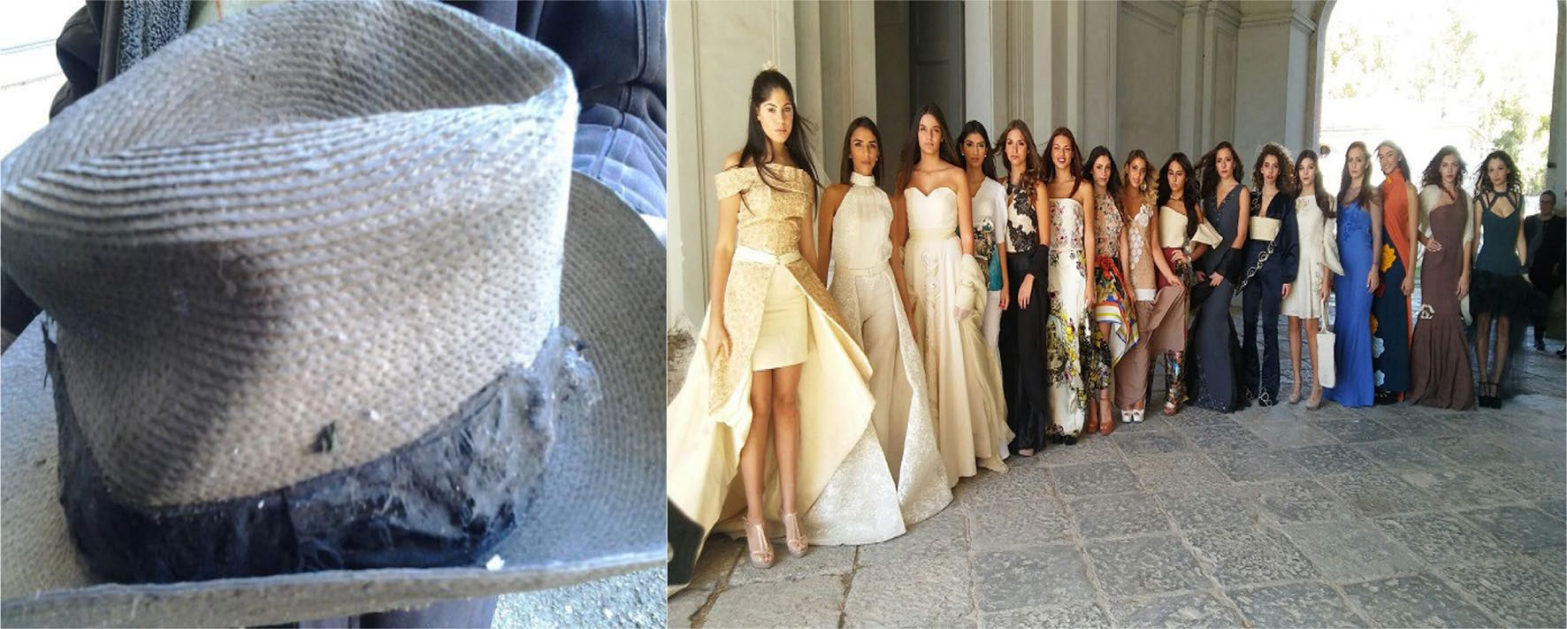
The past and future of hemp in the high fashion sector. On the left, a 1930s borsalino style hemp hat and on the right, the collection of the Maria Corrado Foundation of Marcianise (CE) with hemp garments made in the 1950s by the seamstresses of the hemp center in the Agro-Aversano. The students, in clothes made of hemp, were from the "G. B. Novelli" Institute of Marcianise. The fashion show was presented as part of the event "The return *of hemp in Campania Felix"* which was held on October 8, 2017 at the *Real Sito in Carditello* (CE)

The CNC has been decisive for textile sector development thanks to the fact that it assisted farmers during the agronomic phase by giving Italian varieties seeds such as Eletta Campana and withdrawing at the end of the cultivation cycle the fiber to be allocated to the textile industry. Its dissolution in the 1970s coincided with the abandonment of the textile sector linked to hemp that had been alive for more than a century. Only recently, in 2019, thanks to the new legislation, there were initiatives to restart this sector and was established the National Consortium for the Protection of Hemp: a guarantee for a quality Made in Italy product and the traceability of Italian hemp product in the textile sector (http://www.consorziotutelacanapa.it). In addition, the natural fibers sector, in which hemp is one of the main protagonists, is receiving a lot of interest from economic operators especially from the high fashion sector that considers, in addition to clothing, also accessories (bags and shoes), cosmetics, jewelry and the eyewear industry (see Fig. 5).

New products are being created in this sector such as shoes with natural fibers that allow excellent flexibility by adapting the footprint to the footwear and favoring the so-*called “natural walking”*, that is the correct way to walk. Thanks to the use of hemp fibers mixed with other natural and synthetic fibers, the sole has been made that is adapted to the upper made exclusively of natural fibers, promoting better breathability (Sbordone and Sorrentino 2018). For the textile sector, the factors limiting the chain are the same described for the green building chain, but with the addition of a greater investment required for the construction of modern textile plants.

## Hemp and pharmaceutical industry

Although the greatest economic interest of farmers is addressed to the substances contained in hemp inflorescence that can be used by the pharmaceutical and parapharmaceutical industry, the Italian legislation deliberately has not regulated at moment their use and therefore it would not be possible to trade in and extract medical products from that part of the plant. According to Law 242/2016, the inflorescence is to be considered a productive waste not suitable for economic purposes since the pharmaceutical chain was not considered by the law for using the product derived from industrial hemp. Moreover, non-psychotropic cannabinoids such as cannabidiol (CBD) requests by the medical sector, require a 99% purity (pharmaceutical industry) and at least 80% (phara-pharmaceutical industry) and an absence of contaminants, added to a traceability of the product, which cannot be guaranteed by a protocol of cultivation in the open field. Because of this, the development of the medicinal hemp in pharmaceutical and parapharmaceutical supply chain can only be developed in a controlled environment. The MIPAAF Circular of May 22, 2018 *"Circular on the methods of cultivation and rules of floriculture" also* recognizes the possibility of cultivating inflorescences intended for floriculture, but it does not accept the agamic reproduction. In addition to floriculture, another possible alternative in a controlled environment could be intensive cultivation of hemp roots that are not a source of Δ9-tetrahydrocannabinol (THC), cannabidiol or other known phytocannabinoids, exempting this activity from any possible connection with ludic practices. Root cultivation can be done intensively through aeroponic cultivation system. This agronomic technique without soil provides for the control of all production parameters in a closed circuit thanks to a nutrient solution that is properly sprayed on the roots, allowing the reuse of 80% of the water that is supplied to the plants in the productive circuit. Thanks to this state-of-the-art technique, an extensive carpet of roots is produced, from which, after cutting, biologically active substances are extracted for the medical industry. The production of medicinal substances from hemp roots has a long history of use, in fact they were employed in the making of decoctions and preparations to treat and relieve symptoms caused by a large number of diseases and disorders such as stiffness of the joints and gout. The possibility of using roots is an alternative to the legislative vacuum that prohibits the use of inflorescences, but the development of this new and futuristic supply chain derived from hemp will need, for the future, a new ideotype of plant like the one it can be achieved in an aeroponic cultivation system.

**Fig. 6:**
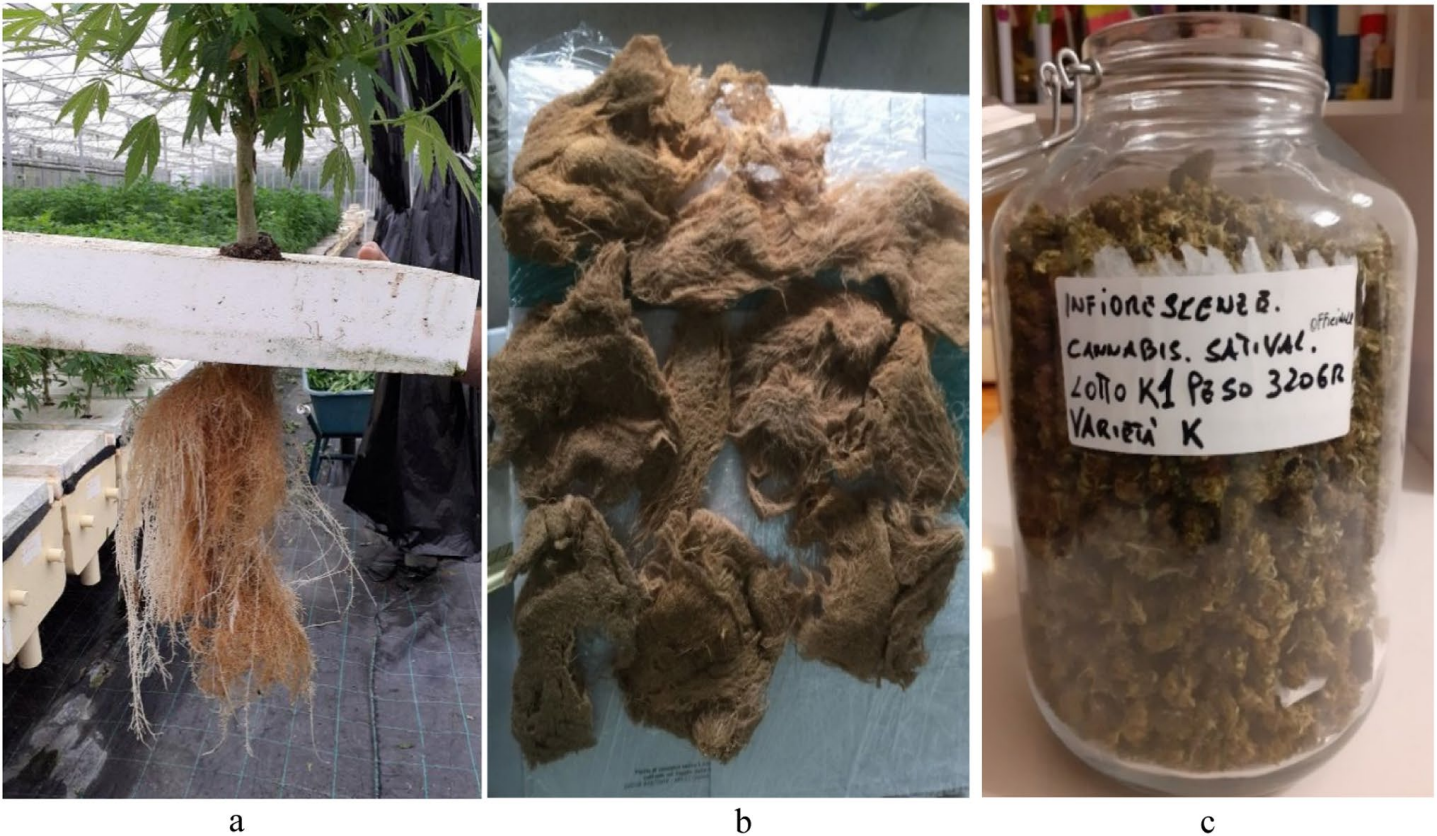
**a** Aeroponics hemp cultivation from Aeroponica Perrotta in Roccarainola (NA)—Italy. **a** Plants produced through aeroponics hemp cultivation; **b** Radical carpet of hemp for the extraction of substances for the pharmaceutical industry; **c** Inflorescences of the lower scaffolding of aeroponics hemp cultivation related to 1 m^2^ of carpet roots

This plant has a very small stem (the plants for textile fiber and for green building have a stem that can reach 3–4 m comparing Fig. 2a vs Fig. 6a) and a very large biomass made entirely of roots and inflorescences with high content of pharmaceutical interest compounds. This type of plant has already been partly developed by an innovative and green start-up such as Aeroponica Perrotta (www.aeroponicaperrotta.it) and is available for an industrialization project for the pharmaceutical industry. Because very low amounts of soil and water are needed for this cultivation technique, there is a higher benefit to utilize it. With this technique, about 360 g of dry roots at 10% humidity is produced per square meter of radical carpet, with a dry matter ratio between roots and inflorescence of 2:1 (Fig. 6b, c). From this surface of roots, a series of compounds of pharmaceutical interest can be extracted, such as triterpenoids and monoterpenes, for example the friedelin in quantities of about 360 mg/kg, a very high value considering the values reported in the literature for a single plant of hemp (Ryz et al. 2017).

Furthermore, it is possible to obtain 720 g of inflorescences per square meter of radical carpet. This means 1 hectare of aeroponic hemp cultivation can be used instead of 12 hectares to obtain the same yield of inflorescences. Thanks to the availability of plants that can reach up to 14% of CBD compared to their dry weight and that even be free from Δ9-tetrahydrocannabinol, it is possible to obtain an amount of CBD equal to about 100 g per m^2^, which is an industrial yield of very high efficiency. None of the varieties of hemp allowed for cultivation in Europe, even if grown with an aeroponic system, can give these yields, considering that they have been selected for other purposes. It is, therefore, necessary in the near future to assess, on the basis of what will be the legislation for this sector, the possibility of patenting new hemp cultivars with high yields in pharmaceutical extracts to be used, through the cultivation in the aeroponic sector, exclusively by the pharmaceutical and parapharmaceutical industries.

## Conclusions

The long and controversial history of *Cannabis sativa* L. that has accompanied the journey of humanity since ancient times is perhaps about to the end because a new and very important chapter is going to be written. Considering that the climate emergency has been defined by the EU as a major priority and no country alone can find a solution, in this regard *Canapa sativa* L. could represent a sort of *green vaccine* worldwide due to its ability to contain and also reduce the damages caused by humans, pointing out a new path of sustainable development in terms of socio-economic advantages and the well-being of the planet.
